# HOXA13 Is Essential for Placental Vascular Patterning and Labyrinth Endothelial Specification

**DOI:** 10.1371/journal.pgen.1000073

**Published:** 2008-05-16

**Authors:** Carley A. E. Shaut, Douglas R. Keene, Lise K. Sorensen, Dean Y. Li, H. Scott Stadler

**Affiliations:** 1 Department of Molecular and Medical Genetics, Oregon Health & Science University, Portland, Oregon, United States of America; 2 Heart Research Center, Oregon Health & Science University, Portland, Oregon, United States of America; 3 Shriners Hospital for Children Research Division, Portland, Oregon, United States of America; 4 Program in Human Molecular Biology and Genetics, University of Utah, Salt Lake City, Utah, United States of America; University of Pennsylvania School of Medicine, United States of America

## Abstract

In eutherian mammals, embryonic growth and survival is dependent on the formation of the placenta, an organ that facilitates the efficient exchange of oxygen, nutrients, and metabolic waste between the maternal and fetal blood supplies. Key to the placenta's function is the formation of its vascular labyrinth, a series of finely branched vessels whose molecular ontogeny remains largely undefined. In this report, we demonstrate that HOXA13 plays an essential role in labyrinth vessel formation. In the absence of HOXA13 function, placental endothelial cell morphology is altered, causing a loss in vessel wall integrity, edema of the embryonic blood vessels, and mid-gestational lethality. Microarray analysis of wild-type and mutant placentas revealed significant changes in endothelial gene expression profiles. Notably, pro-vascular genes, including *Tie2* and *Foxf1*, exhibited reduced expression in the mutant endothelia, which also exhibited elevated expression of genes normally expressed in lymphatic or sinusoidal endothelia. ChIP analysis of HOXA13–DNA complexes in the placenta confirmed that HOXA13 binds the *Tie2 and Foxf1* promoters in vivo. In vitro, HOXA13 binds sequences present in the *Tie2* and *Foxf1* promoters with high affinity (K_d_ = 27–42 nM) and HOXA13 can use these bound promoter regions to direct gene expression. Taken together, these findings demonstrate that HOXA13 directly regulates *Tie2* and *Foxf1* in the placental labyrinth endothelia, providing a functional explanation for the mid-gestational lethality exhibited by *Hoxa13* mutant embryos as well as a novel transcriptional program necessary for the specification of the labyrinth vascular endothelia.

## Author Summary

Defects in placental development are a common cause of mid-gestational lethality. Key to the placenta's function is its vascular labyrinth, a series of finely branched vessels that facilitate the efficient exchange of gases, nutrients, and metabolic waste between the maternal and fetal blood supplies. In this study, we identify a novel role for the transcription factor HOXA13 in formation of the placental vascular labyrinth. In the absence of HOXA13 function, labyrinth vessel branching and endothelial specification is compromised, causing mid-gestational lethality due to placental insufficiency. Analysis of the genes affected by the loss of HOXA13 function revealed significant reductions in the expression of several pro-vascular genes, including *Tie2* and *Foxf1*. Analysis of the *Tie2* and *Foxf1* promoters confirmed that HOXA13 binds sites present in each promoter with high affinity in the placenta, and *in vitro,* HOXA13 can use these bound sequences to regulate gene expression. These results suggest that *Tie2* and *Foxf1* are direct transcriptional targets of HOXA13 in the developing placental labyrinth, providing a novel transcriptional pathway to consider when examining pathologies of the placenta and placental insufficiency, as well as the evolutionary mechanisms required for the emergence of the vascular placenta in eutherian mammals.

## Introduction

For placental mammals, fetal development is contained in an intrauterine environment where the efficient exchange of oxygen, nutrients, and metabolic waste between the maternal and fetal blood supplies is facilitated by the placenta. Central to the placenta's function is its vascular labyrinth, a juxtaposed series of finely-branched blood vessels and trophoblasts that regulate nutrient and waste exchange while maintaining the separation of the maternal and fetal blood supplies [1]. After implantation, labyrinth vascularization proceeds from the allantois, where angiogenic and vasculogenic processes promote the formation of a dense, highly arborized vascular bed [2]–[7]. The formation of the labyrinth vascular bed requires many of the same signals controlling embryonic vascular development including: VEGF and its associated receptors FLT1, FLK1, and NEUROPILIN-1, as well as ANG-1 and ANG-2 and its receptor TIE-2 [8]–[16]. Interestingly, while loss of function studies clearly demonstrate that transcription factors such as: TBX4, CDX2, CDX4, HAND1, DLX3, FOXF1, and CITED2 are required for placental development, the target genes regulated by these proteins in the developing placenta are largely undefined [3], [17]–[26]. In this report, we describe a novel role for HOXA13 in the developing placenta and identify both direct and indirect targets of HOXA13 functioning in the placental labyrinth endothelia. In the absence of HOXA13 function, labyrinth endothelial cell morphology, vessel branching, and vessel integrity are compromised, a consequence we attribute to a loss in the regulation of several essential pro-vascular genes. Chromatin immunoprecipitation of the HOXA13-DNA complexes confirmed that HOXA13 directly associates with the *Tie2* and *Foxf1* promoters in vivo in the developing placenta. Quantitation of HOXA13's affinity for these promoter regions confirmed that HOXA13 binds these regions with high affinity and can utilize these bound DNA sequences to facilitate gene expression in vitro. Together these findings reveal a novel temporal and spatial domain for HOXA13 function in the developing embryo and identify a key transcriptional hierarchy necessary for the development of the placental vascular labyrinth.

## Results

### *Hoxa13* Is Expressed Throughout Placental Labyrinth Development

**Figure 1:**
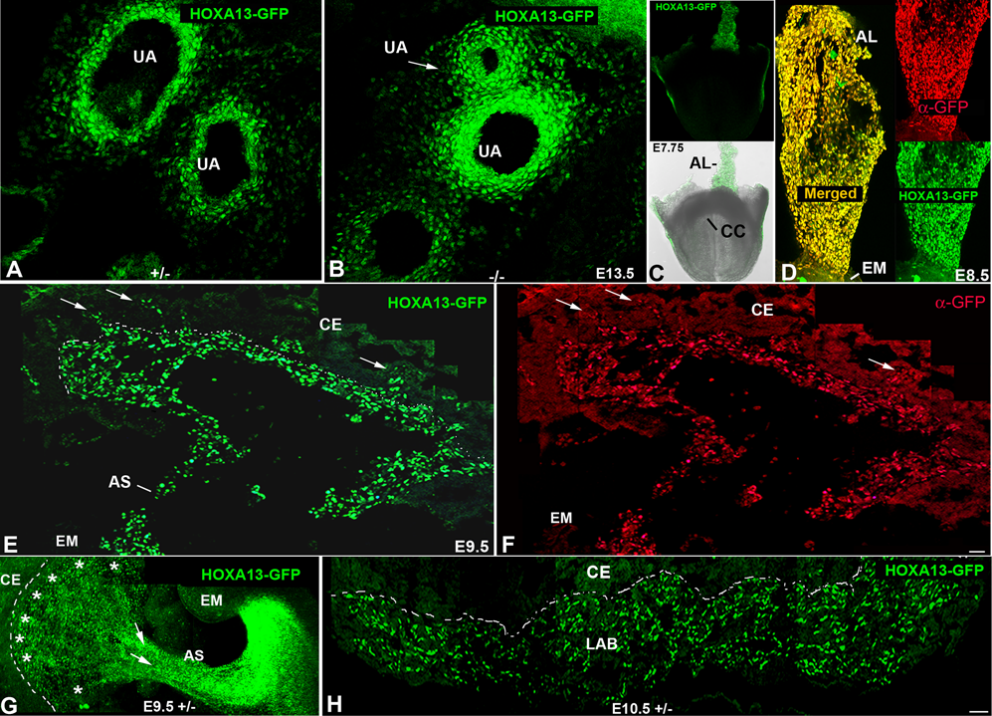
Early expression of *Hoxa13* in the allantois and its derivatives. (A, B) HOXA13 is expressed in the umbilical arteries which partially stenose (white arrow) in *Hoxa13* homozygous mutants. (C) Fluorescent and bright field image of an E7.75 embryo expressing HOXA13-GFP. AL = allantois, CC = cardiac crescent. (D) HOXA13-GFP is expressed throughout the allantois at E8.5. Red signal = detection of the HOXA13-GFP fusion protein using a GFP antibody (denoted as α-GFP). Green signal indicates detection of the endogenous HOXA13-GFP fusion protein. Yellow signal (Merged) indicates the co-localization of the detected HOXA13-GFP protein and the α-GFP immuno-positive cells, confirming the detected green fluorescence to be derived from the mutant HOXA13-GFP fusion protein throughout the allantois. AL = allantois; EM = embryo proper. (E, F) Cryosection of an E9.5 placenta. Green signal indicates detection of the HOXA13-GFP fusion protein in the developing labyrinth region; red signal indicates detection of the HOXA13-GFP fusion protein using a GFP antibody (denoted as α-GFP). Note the absence of HOXA13-GFP expression in the chorionic ectoderm (CE). Arrows denote sites of microvessel genesis. Dashed line represents chorionic plate. AS = allantoic stalk. (G) Sagittal section of an E9.5 embryo and developing placenta. Note that HOXA13-GFP expression is maintained in the allantoic stalk as it contributes to the developing allantoic vessels (arrows) as well as the developing chorionic plate vessels (asterisks). Dashed line denotes the developing chorionic plate. CE = chorionic ectoderm, EM = embryo proper, AS = allantoic stalk. (H) At E10.5, HOXA13 expression is maintained in the developing labyrinth (LAB), whereas little or no expression is detected in the chorionic ectoderm (CE). Bars are 25 µm.

**Figure 2:**
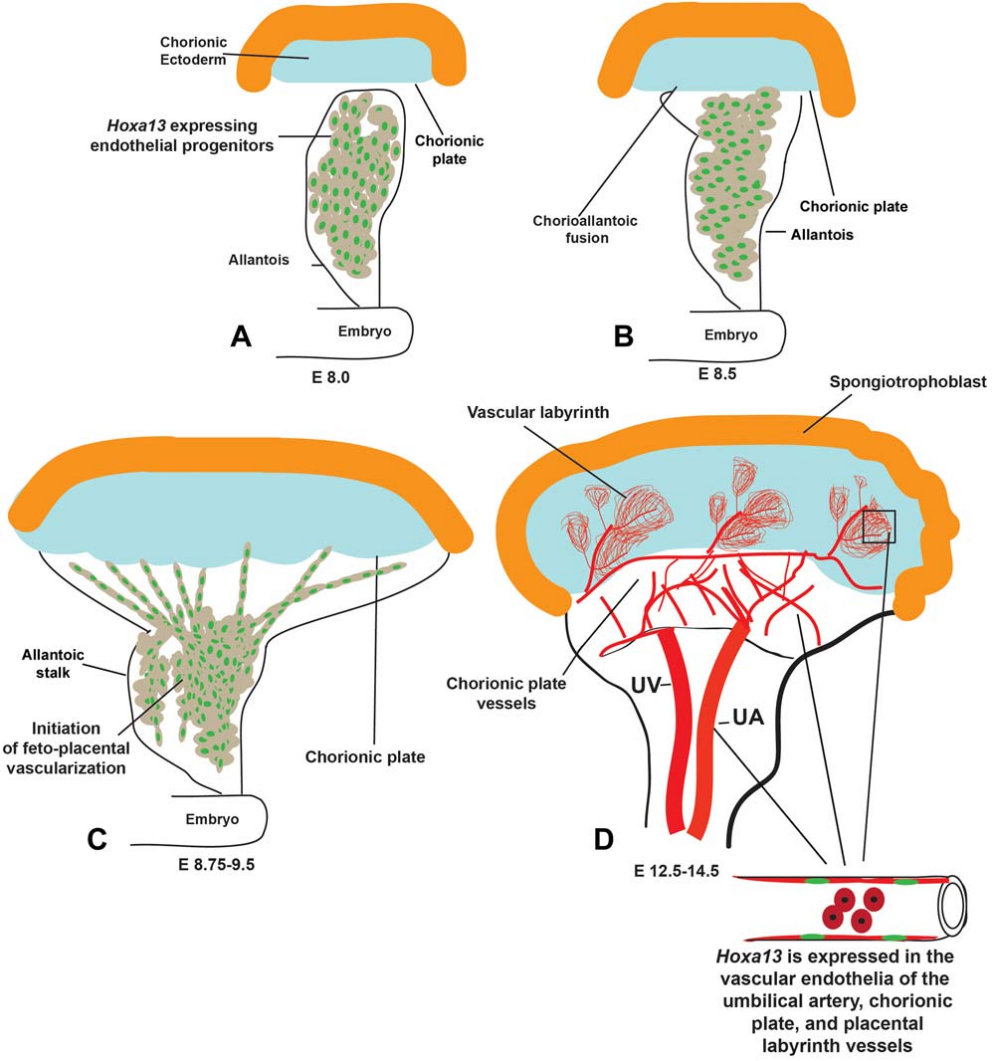
Sites of HOXA13 expression during placental labyrinth development. (A) HOXA13 is expressed in the endothelial progenitors in the E8.0 allantois. (B) HOXA13 expression is maintained in the endothelial progenitors during chorioallantoic fusion at E8.5. (C) Between E8.75 and E9.5, the HOXA13-expressing endothelial progenitors contribute to the developing feto-placental vessels, which mature to form the umbilical artery (UA), chorionic plate vessels, and the vessels contributing to the placental labyrinth (D). HOXA13 is not expressed in the developing umbilical vein (UV).

Among the 39 murine Hox genes, only mutations in *Hoxa13* cause mid-gestational lethality from embryonic day (E) 11–15.5 [27]–[29]. Initially, this phenotype was attributed to premature stenosis of the umbilical arteries [29]. Extensive analysis of the umbilical artery (UA) defect in *Hoxa13* homozygous mutants revealed that only one of the two UAs exhibited complete stenosis from E11.5–15.5 (Figure 1A and 1B). This finding prompted the hypothesis that an additional defect must be contributing to the mid-gestational lethality. Because malformations of the heart and placenta are the most commonly cited reasons for mid-gestational lethality, we first examined whether *Hoxa13* is expressed in the developing heart or placenta [30]–[34]. No HOXA13 expression was detected in the cardiac crescent; however, the earliest component of the placenta, the allantoic bud mesoderm, strongly expressed HOXA13 from E7.75 and maintained expression in the maturing allantois and developing labyrinth microvessels at E8.5 and E9.5 respectively (Figures 1C–1G and 2). At E10.5, HOXA13 expression was readily detected in the developing placental labyrinth (Figures 1H and 2) whereas the chorionic ectoderm exhibited little or no HOXA13 expression from E9.5 to E10.5 (Figures 1E–1H, 2). To note, the timing and occurrence of chorioallantoic fusion was unaffected by the loss of HOXA13 function (data not shown).

### HOXA13 Functions in the Labyrinth Vascular Endothelia

**Figure 3 pgen-1000073-g003:**
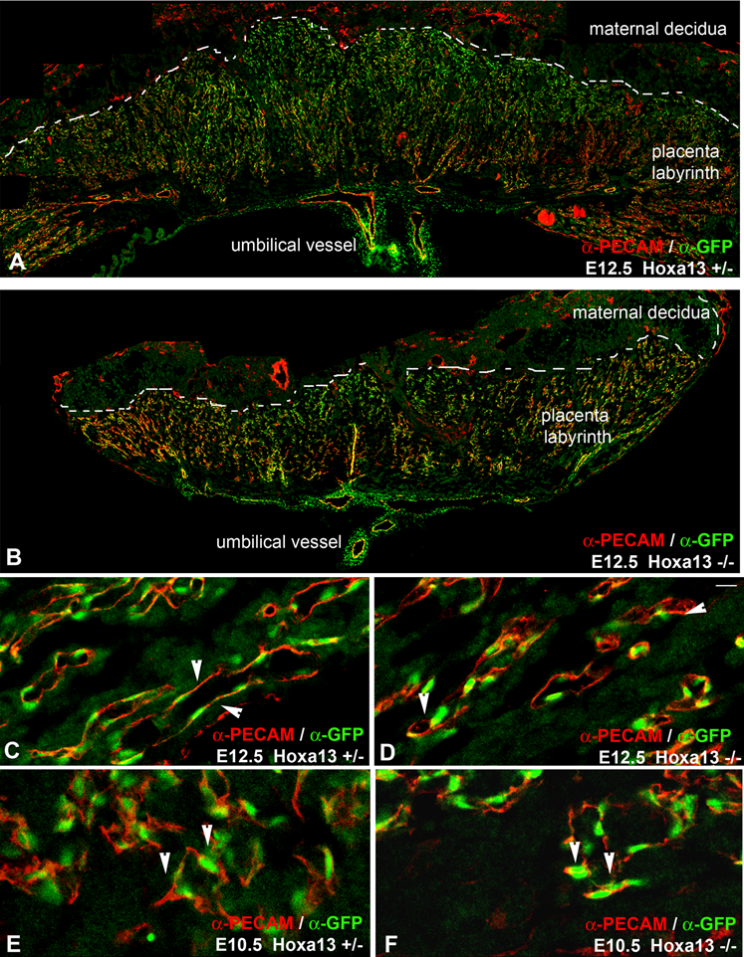
HOXA13 is expressed in the placental labyrinth vascular endothelia. (A, B) Analysis of HOXA13 expression (green) in heterozygous control and homozygous mutant placentas at E12.5 reveals extensive expression throughout the vascular labyrinth. (C) HOXA13 is co-expressed with PECAM-1 in the labyrinth endothelia, which exhibit an elongated morphology in heterozygous controls (arrowheads). (D) E12.5 homozygous mutant labyrinth endothelia also express HOXA13 (green signal) and PECAM-1 (red signal), but maintain a rounded morphology (arrowheads). (E, F) Analysis of labyrinth vessels at E10.5 reveals co-localization of HOXA13 (green signal) and PECAM-1 (red signal) to the undifferentiated endothelia (arrowheads), which do not exhibit an elongated vascular morphology. Bar is 10 µm for (C–F).

**Figure 4:**
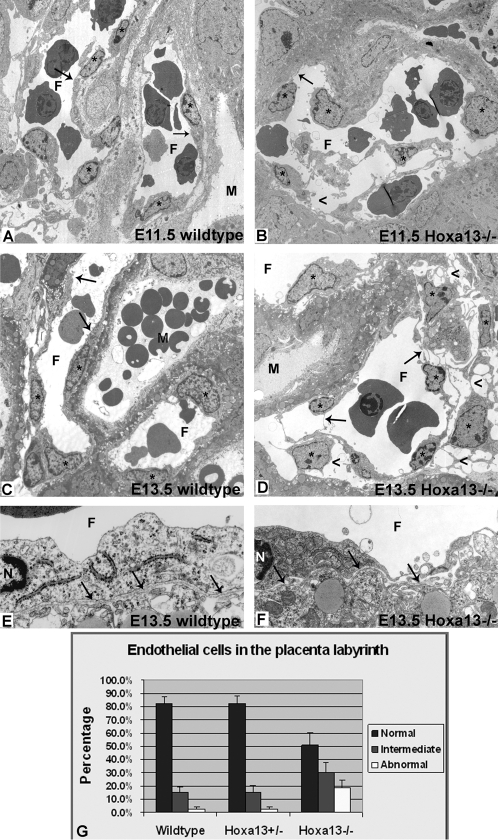
Endothelial cell morphology is affected in the placental labyrinth of *Hoxa13* homozygous mutants. (A, B) Transmission electron microscopy (TEM) reveals the initial elongation of the wild-type EC in the developing labyrinth vessels, whereas homozygous mutant littermates (B) exhibit rounded endothelia with attenuated cell bodies. Asterisks denote the endothelial cells; arrows depict the EC bodies in wild-type and mutant vessels. (C, D) Wild-type controls exhibit a mature elongated endothelial cell morphology by E13.5 (arrows), whereas homozygous mutant littermates (D) exhibit a severe loss in the elongated morphology (arrows), causing edema in the surrounding placental tissues (arrowheads). F = fetal vessel lumen; M = maternal space. (E, F) No differences in basement membrane (arrows) ultrastructure were detected in the labyrinth vessels between wild-type and homozygous mutant embryos, confirming that the loss of HOXA13 function is directly affecting endothelial morphology and function. F = fetal vessel lumen; N = endothelial cell nucleus. (G) Quantitative analysis of multiple labyrinth vessel TEM micrographs revealed that nearly 50% of the homozygous mutant endothelia exhibited an intermediate or severely abnormal morphology compared to wild-type or heterozygous mutant controls.

To determine the identity of the cells expressing HOXA13 in developing labyrinth, we examined whether these cells co-express the endothelial marker PECAM-1 (Figure 3) [35]–[37]. Characterization of HOXA13 and PECAM-1 expression confirmed that only the cells expressing PECAM-1 (cell surface) also express HOXA13 (nucleus), suggesting that HOXA13 is functioning in the labyrinth vascular endothelial cells (EC) (Figure 3A–3F). HOXA13 expression was not detected in the placental trophoblasts (data not shown). Interestingly, the elongated morphology normally attributed to the labyrinth vascular endothelia was also affected in homozygous mutants, which appeared rounded compared to controls in the E12.5 labyrinths (Figure 3C and 3D). To determine the onset of the EC phenotype, we characterized the labyrinth vessels at E10.5 (Figure 3E and 3F). At E10.5, the EC in both the heterozygous control and homozygous mutant labyrinths exhibited only the rounded EC morphology (Figure 3E and 3F). This result suggests that the vascular specification of the EC, as indicated by their elongated morphology, occurs between E10.5 and E12.5 and denotes when the loss of HOXA13 function phenotype first manifests in the developing labyrinth EC (Figure 3C–3F). Close examination of the affected EC using transmission electron microscopy confirmed the timing of the onset of this phenotype as wild type EC exhibited lengthening of the cell body as early at E11.5 whereas homozygous mutant EC exhibited shortened cell bodies that lacked uniform contact with the underlying vessel walls (Figure 4A–4D). Finally, consistent with the reduction in the EC cell body was the loss of vessel wall integrity in the E11.5 and E13.5 mutant vessels, resulting in extracellular edema between mutant labyrinth vessels and the underlying syncytiotrophoblasts, while edema was not detected in the wild type labyrinths (Figure 4A–4D).

No gross morphological defects were observed in the basement membranes between the EC and the syncytiotrophoblast (Figure 4E and 4F). Quantitation of the affected labyrinth EC in the homozygous mutants revealed that nearly 50 percent exhibited an intermediate or grossly affected morphology compared to only 12 percent for age-matched controls (Figure 4G). Finally, the affected EC did not appear apoptotic as the nuclei lacked a pyknotic phenotype as well as TUNEL positive staining (Figure 4 and unpublished data).

### Labyrinth Vascular Branching and Size Are Reduced in *Hoxa13* Homozygous Mutants

As the umbilical vessels cross the chorionic plate, they exhibit non-sprouting angiogenesis parallel to the chorionic plate to produce the chorionic plate vessels, followed by additional EC migration and branching angiogenesis into the labyrinth to create a complex vascular tree (see Figure 2). In the murine placental labyrinth, these vascular branches are interconnected, signifying that intussusceptive angiogenesis and vessel fusion also play a role in their angiogenic remodeling [38],[39]. Recognizing that EC mediate many of the angiogenic processes necessary for vessel remodeling and branching, we hypothesized that defects in the mutant labyrinth EC would affect vessel branching, which would compromise the capacity of this structure to sustain embryonic survival (Figures 2–4) [40]–[47]. To best visualize the branched vasculature within the labyrinth, we utilized whole tissue immunohistochemistry using the PECAM-1 antibody and hemisected placentas.

**Figure 5:**
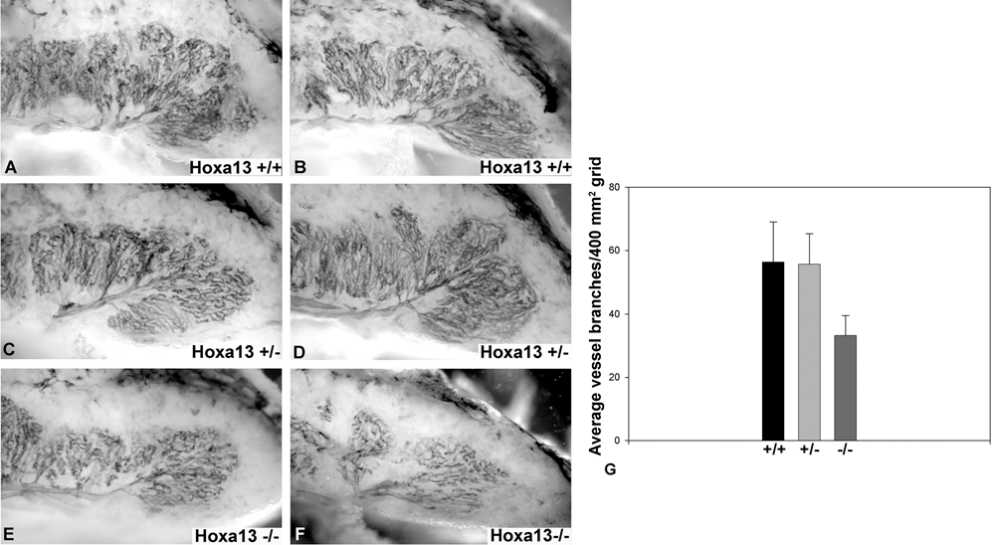
Labyrinth vessel branching is reduced in *Hoxa13* homozygous mutants. (A–D) PECAM-1 immunostaining of E13.5 hemisected placentas revealed extensive branching of the primary labyrinth vessel in wild-type and heterozygous mutants, whereas homozygous mutant littermates exhibited poor branching of the primary labyrinth vessels (E, F). (G) Quantitation of the labyrinth vascular branches confirmed that the homozygous mutants labyrinths contained an average of 33 branches per 400 mm^2^ grid, whereas wild-type and heterozygous mutant controls contained an average of 56 and 55 branches, respectively, in the same unit area. Bars represent the standard deviation of six independent assessments.

**Figure 6:**
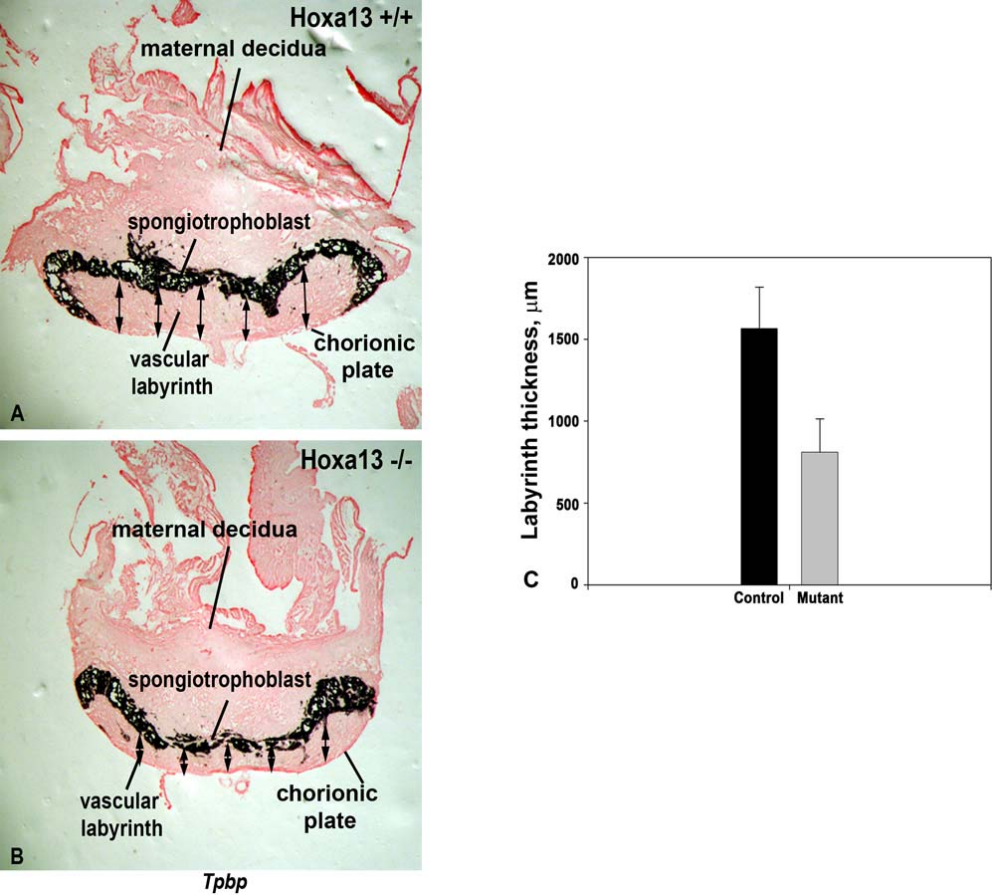
The vascular labyrinth region is thinner in *Hoxa13* homozygous mutant placentas. (A), (C) Measurements of the E12.5 labyrinth, which is defined as the region underlying the spongiotrophoblast marker, *Tpbp*, revealed an average labyrinth thickness of nearly 1,600 µm in *Hoxa13* control embryos. (B, C) Parallel analyses of age-matched *Hoxa13* homozygous mutants confirmed that the labyrinth thickness is reduced to an average thickness of 800 µm. Arrows represent the sites measured on each labyrinth section from the spongiotrophoblast to the chorionic plate to determine labyrinth thickness using the NIH Image J software. Error bars represent the standard deviation of 16 different labyrinth sections measured at 5 independent points for each sample.

Indeed, while the initial vascular invasion of the chorionic plate at E10.5 appears normal in *Hoxa13* homozygous mutants, there is a qualitative reduction in the level of PECAM-1 staining of the labyrinth vasculature as early as E11.5 (Figure S1). Quantitation of the vascular branches in the mutant and control placental labyrinths at E13.5 confirmed that the number of branches is reduced in the *Hoxa13* homozygous mutants which exhibited 33 (±6.3) branches per 400 mm^2^ grid analyzed, whereas wild type and heterozygous mutant controls contained 56 (±12.7) and 55 (±9.7) vessel branches respectively (Figure 5). Qualitatively, the reduction in labyrinth vascularity in homozygous mutants was persistent throughout labyrinth development which is complete by E14.5, suggesting that decreased vessel branching is phenotypic of the loss of HOXA13 function rather than a delay in the labyrinth maturation (Figures 3, 5, and S1) [48]. Next, because changes in vessel branching can also affect the overall size of the labyrinth, we examined whether the labyrinth region was smaller in *Hoxa13* mutant placentas. Analysis of labyrinth sections taken from E13.5 wild type and *Hoxa13* homozygous mutants confirmed that the mutant labyrinths were nearly half the thickness as their age-matched controls (800±200 µm vs. 1600±250 µm) (Figure 6).

**Figure 7:**
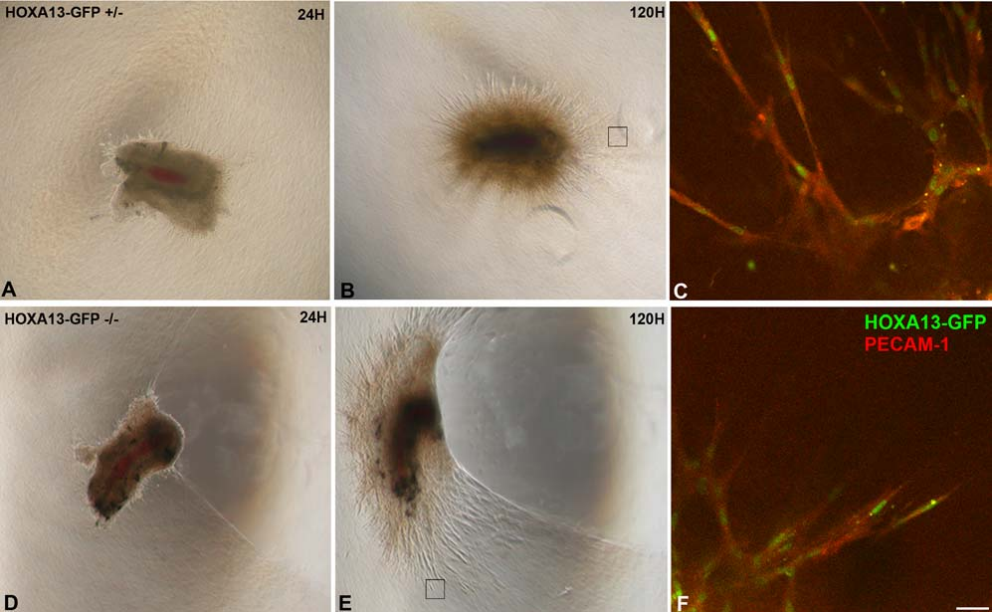
Analysis of endothelial cell migration and neovascularization in cultured placental primary arteries. (A, B), (D, E) Arterial sections from heterozygous control and homozygous mutants exhibit robust neovascularization and microvessel formation in vitro. Black boxes denote the sites examined by confocal microscopy to visualize migrating endothelia participating in microvessel formation. (C), (F) The loss of HOXA13 function does not affect the migration and contribution of HOXA13-expressing endothelia (green nuclei) to the developing microvessels. Note that the migrating endothelia co-express PECAM-1 (red signal). Bar is 25 µm.

Since defects in endothelial cell migration can also contribute to perturbations in vessel branching, we examined whether *Hoxa13* mutant endothelia were competent to migrate and participate in *de novo* angiogenesis (Figure 7). A comparison of mutant and heterozygous control cultured arterial sections revealed comparable levels of neo-vessel production after five days of growth (Figure 7A and 7B and 7D and 7E). Characterization of the cells contributing to the neo-vessels revealed strong HOXA13 expression in the PECAM-1 positive endothelia in both heterozygous and homozygous mutant (Figure 7C and 7F). This result suggests that endothelial cell migration is not affected by the loss of HOXA13 function in the *in vitro* angiogenesis assay.

**Figure 8:**
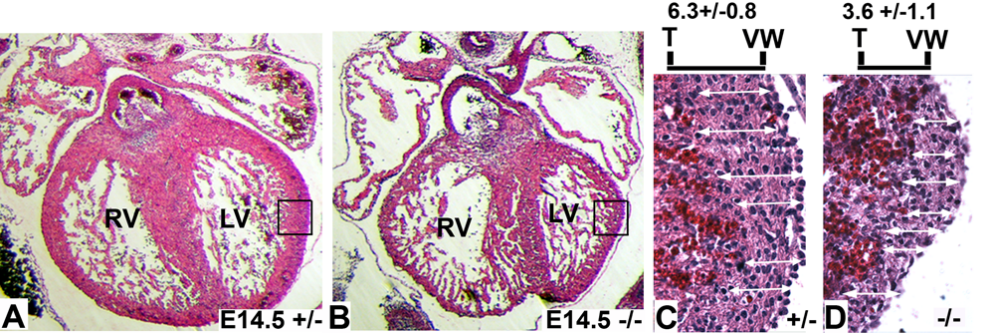
Analysis of ventricular wall thickness in Hoxa13 mutants. (A, B) Analysis of hematoxylin and eosin–stained sections (7 µm) from heterozygous control and homozygous mutant hearts at E14.5 embryos reveal qualitatively thicker ventricular walls in the heterozygous control embryos. RV = right ventricle; LV = left ventricle. Boxes represent the enlarged regions shown in (C) and (D). (C, D) Quantitative analysis of the left ventricular wall thicknesses revealed homozygous mutants possess an average of 3.6 (±1.1) cells per linear assessment versus 6.3 (±0.8) cells in heterozygous controls, confirming that the E14.5 Hoxa13 homozygous mutants possess a thinner left ventricular wall compared to age-matched heterozygous controls. White arrows depict the sites assessed for ventricular wall thickness using the hematoxylin-stained nuclei to determine cell number. The measurements were taken from the trabecular wall (T) to the outer ventricular wall (VW) for each assessment.

Taken together, these results suggest that placental insufficiency caused by decreased labyrinth vascularity and size may be causing the mid-gestational lethality associated with the loss of HOXA13 function. To test this hypothesis we examined the developing heart, an organ that does not express *Hoxa13* but is severely affected by placental insufficiency [28], [29], [49]–[53]. Analysis of E14.5 hearts from heterozygous control and homozygous mutants revealed a substantial thinning of the right and left ventricular walls in homozygous mutants (Figure 8). Quantitation of the left ventricular wall thicknesses confirmed nearly a 43% reduction in wall thicknesses in homozygous mutants which contained an average of 3.6 (±1.1) cells per wall section measurement versus 6.3 (±0.8) cells in the comparable sections of age-matched heterozygous controls (Figure 8C and 4D).

### The Loss of HOXA13 Function Causes Mis-Expression of Pro-Vascular and Non-Capillary Endothelial Genes

To identify candidate HOXA13 target genes which are functioning in the placental labyrinth, we performed microarray analysis on E13.5 labyrinth tissues. Twelve microarray hybridizations (6 mutant; 6 wild type) were performed using independent isolates of placental labyrinth total RNA. Statistical analysis of the gene expression signals detected by the microarray probe sets identified significant reductions in pro-vascular gene expression (FDR≤0.05) including: *CD36, necdin, Enpp2, Adrb1, Tie2, Foxf1, Neuropilin-1, Magel-2*, and *Caveolin-1* (Table 1). Interestingly, the homozygous mutant placentas also exhibited significant over-expression of several genes normally expressed in sinusoidal and lymphatic endothelia including: *Lyve-1, Igfbp3, Selenbp1, Bmp1*, and *Ednrb* (Table 2) [54]–[58]. Finally, genes expressed in specific trophoblast lineages such as: *gcm1* (chorionic trophoblast), *esx1* (labyrinth trophoblasts), *tpbp* (spongiotrophoblast), *Id1* (chorionic trophoblast), and *Id2* (chorionic trophoblast) exhibited no significant changes in expression between control and *Hoxa13* mutant placentas (FDR≤0.05) (Figure 6 and Table 3) [59]–[63].

**Table 1:** Microarray and qRT-PCR analysis of gene transcription comparing mutant and wild-type tissue.

Affymetrix Array Probe ID	Gene	GO: Molecular Function/Biological Process	qRT-PCR Primers (5′ - 3′)	MUT/WT Fold Change	
				Whole Placenta Microarray (FDR)	Endothelial Cell–Specific qRT-PCR
1421201_a_at	*Trophinin*	Cell adhesion/Cell growth and organization	GAACCCACGACCAGAACC – For GCAAAATGGCCACATCTC – Rev	−2.2 (0.00)	−5.4
1448136_at	*Enpp2*	Hydrolase and nucleotide diphosphatase activity/Chemotaxis; cell motility	CCGACCTGACAATGATGAGA – For AAATCCAAACCGGTGAGATG – Rev	−2.3 (0.00)	−23.1
1417217_at	*Magel-2*	Protein binding/Regulation of transcription	AACGCTTTGGTGCAGTTTCT – For CTTAGTGTTGGCACGGTTGA – Rev	−2.9 (0.00)	−98.9
1449145_a_at	*Caveolin-1*	Protein binding/Vasoconstriction; vasodilation; EC proliferation	GGGAACAGGGCAACATCTAC – For AACACGTCGTCGTTGAGAT – Rev	−1.9 (0.00)	−3.6
1423420_at	*Adrb1*	Receptor activity/Blood pressure regulation; vasodilation	GCTGATCTGGTCATGGGATT – For AAGTCCAGAGCTCGCAGAAG – Rev	−1.9 (0.00)	−8.0
1418788_at	*Tie2 (Tek)*	Receptor activity/Regulation of angiogenesis and cell migration; cell adhesion	TGAGGACGCTTCCACATTC – For CAACAGCACGGTATGCAAGT – Rev	−1.6 (0.00)	−2.2
1429379_at	*Lyve1 (Xlkd1)*	Receptor activity; hyaluronic acid binding/Cell adhesion	AGCCAACGAGGCCTGTAA – For CACCTGGGGTTTGAGAAAAT – Rev	+3.6 (0.00)	+4.4
1450883_a_at	*CD36*	Receptor activity/Cell adhesion; fatty acid and lipid metabolism and transport	GAGTTGGCGAGAAAACCAGT – For GTCTCCGACTGGCATGAGA – Rev	−2.28 (0.01)	−2.9
1418084_at	*Neuropilin-1*	Receptor activity; VEGF receptor activity/Angiogenesis; cell migration	TGTCCTGGCCACAGAGAAG – For CCAGTGGCAGAATGTCTTGT – Rev	−1.5 (0.02)	−7.4
1435382_at	*Necdin*	Transcription factor/Cell growth and migration	TGGTACGTGTTGGTGAAGGA – For AACACTCTGGCGAGGATGAC – Rev	−1.8 (0.00)	−7.7
1434939_at	*FoxF1*	Transcription factor/Vasculogenesis; organ development; ECM organization	GCAGCCATACCTTCACCAA – For GCCATGGCATTGAAAGAGA – Rev	−1.3 (0.03)	−2.9

**Table 2:** Loss of HOXA13 function causes increased sinusoidal and lymphatic endothelial gene expression.

Affymetrix Array Probe ID	Gene	Molecular Function/Biological Process	Fold Change MUT/WT (FDR)
			Whole Placenta Microarray
1429379_at	*Lyve-1/Xlkd1*	Hyaluronate receptor: maintenance of hyaluronan status in lymphatic and sinusoidal endothelia [101]	3.60 (0.00)
1423062_at	*Igfbp3*	Igf Binding protein: maintenance of sinusoidal endothelial phenotype [57]	1.86 (0.00)
1450699_at	*Selenbp1*	Selenium binding protein: maintenance of selenium status and sinusoidal endothelial phenotype [53]	1.52 (0.00)
1427457_a_at	*Bmp1*	Metalloprotease: cleaves prolactin to inhibit angiogenesis [54]	1.36 (0.00)
1426314_at	*Ednrb*	*Endothelin* receptor: promotes sinusoidal endothelial function [56]	1.19 (0.01)

**Table 3:** Loss of HOXA13 function does not affect trophoblast gene expression.

Affymetrix Array Probe ID	Gene	Molecular Function/Biological Process	Fold Change MUT/WT (FDR)
			Whole Placenta Microarray
1420601_at	*Gcm1*	Chorionic trophoblast transcription factor [86]	−1.11 (0.19)
1415808_at	*Tpbpa*	Spongiotrophoblast	−1.04 (0.46)
		Possible function in lysosomal protein degradation [60],[62]	
1425895_a_at	*ID1*	Chorionic trophoblast transcription factor [59]	−1.09 (0.20)
1422537_a_at	*ID2*	Chorionic trophoblast transcription factor [58]	−1.05 (0.45)
1420602_a_at	*Esx1*	Labyrinth trophoblast transcription factor [62]	−1.12 (0.12)

**Figure 9:**
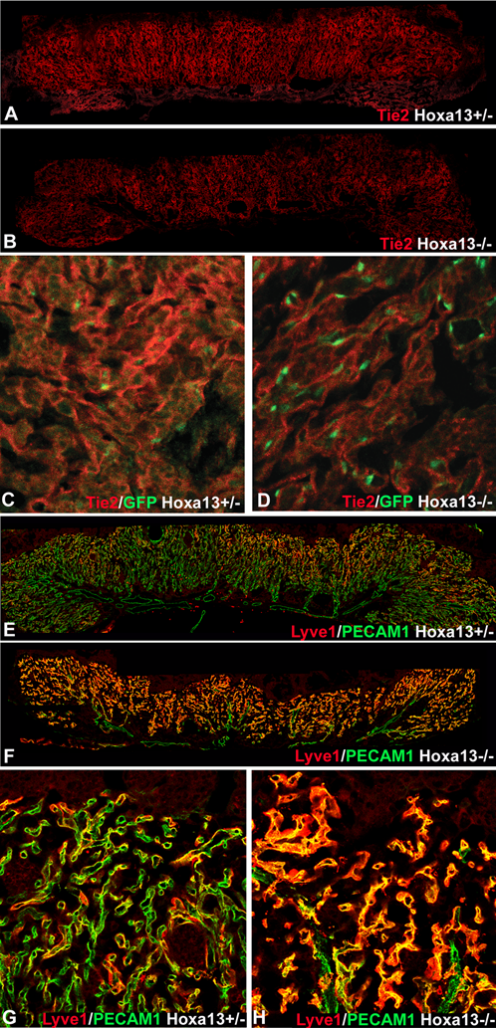
TIE-2 and LYVE-1 are co-expressed with HOXA13 in the labyrinth vascular endothelia and exhibit altered expression in E13.5 homozygous mutants. (A, B) Immunohistochemical analysis of TIE2 expression (red signal) in the placental labyrinth reveals reduced levels in E13.5 *Hoxa13* homozygous mutants compared to heterozygous controls. (C, D) Higher magnification image of the placental labyrinth confirms that TIE2 (red signal) is co-expressed with HOXA13 (green nuclear signal) in the labyrinth vascular endothelia. (E, F) Immunohistochemical analysis of LYVE-1 expression (red signal) in the placental labyrinths reveals elevated levels of LYVE-1 in E13.5 *Hoxa13* homozygous mutants compared to heterozygous controls. (G, H) Analysis of LYVE-1 expression at higher magnification (red signal) confirms that the PECAM-1–positive endothelial cells (green signal) co-express LYVE-1 and that LYVE-1 expression is elevated in the labyrinth vasculature of Hoxa13 homozygous mutants.

Next, to validate that the genes detected by the microarray analysis were mis-expressed in the affected EC, we performed quantitative real-time PCR (qRT-PCR) using total RNA derived from affinity purified vascular labyrinth EC. In all cases, the mis-expression trend determined by the microarray analysis (increased or decreased in mutant placentas) was conserved (Table 1). Moreover, the enrichment of the EC also caused an increase in the detected fold-change differences between wild type and homozygous mutant EC, a finding consistent with an endothelial-specific expression pattern or function for the affected genes. Immunohistochemical analysis of TIE2, LYVE-1, NEUROPILIN-1, and ENPP2 confirmed the altered EC-specific expression levels detected by microarray and qRTPCR (Figures 9 and S1, Table 1, and unpublished data). In particular, the pro-vascular receptor tyrosine kinase, TIE2, was noticeably reduced in the mutant labyrinth EC which also express HOXA13 at E10.5–13.5 (Figures 9A–9D and S1).

### HOXA13 Directly Regulates *Tie2* and *Foxf1*

**Figure 10:**
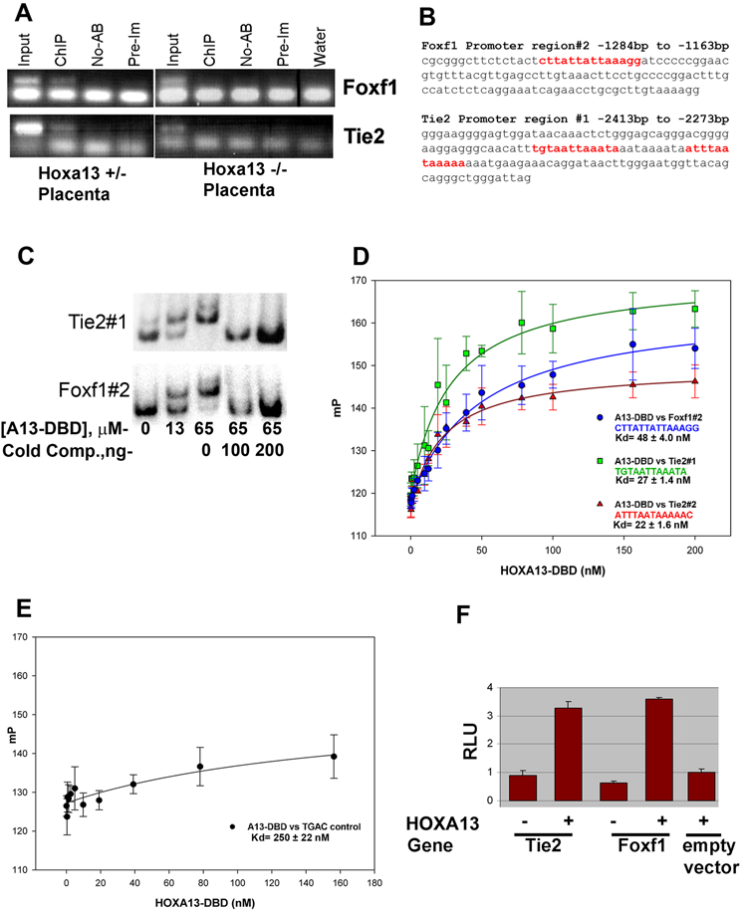
HOXA13 binds to gene-regulatory regions in *Tie2* and *Foxf1* to facilitate gene expression. (A). Left panel: Chromatin immunoprecipitation (ChIP) of heterozygous control placental chromatin using a HOXA13 antibody identified DNA sequences present in the *Tie2* and *Foxf1* promoter regions and confirms that HOXA13 associates with these sequences *in vivo*. Right panel: Parallel assays using *Hoxa13* homozygous mutant placentas failed to enrich for the same DNA regions, suggesting that HOXA13's DNA binding function, which is absent in the homozygous mutant protein, is necessary for in vivo association at the *Tie2* and *Foxf1* loci. Input indicates positive control confirming the presence of the *Tie2* and *Foxf1* DNA elements in both heterozygous and homozygous mutant chromatin samples. ChIP = + HOXA13 antibody; No-AB = no antibody negative control; Pre-Im = IgG control rabbit pre-immune sera; Water = no DNA PCR control (B). Sequence analysis revealed HOXA13 binding sites (red text) in the ChIP-positive PCR amplification products for both *Foxf1* and *Tie2*. (C) EMSA analysis of the ChIP-positive PCR amplification products confirms HOXA13 can bind these sites in a concentration-dependent manner. A13-DBD = HOXA13 DNA binding domain peptide; Cold Comp.  = the *Tie2* or *Foxf1* binding sites lacking radioactive labeling. (D) The HOXA13 DNA binding domain binds the DNA sequences present in the ChIP-positive region with high affinity exhibiting a K_d_ of 48±4 nM for the *Foxf1* region and 27±1.4 nM and 22±1.6 nM for the two sequences present in *Tie2*. Error bars denote the standard error for the averaged millipolarization values at each HOXA13-DBD concentration. The DNA sequences of the *Tie2* or *Foxf1* promoter regions used in the assay are denoted by the color text. (E) Conversion of the Tie2 or Foxf1 binding sites to the sequence TGAC ablates the affinity of the HOXA13 DNA binding domain for these gene-specific promoter sequences. Error bars denote the standard error for the averaged millipolarization values at each HOXA13-DBD concentration. (F) *In vitro* assessment of the 140 bp *Tie2* and 121 bp *Foxf1* ChIP fragments confirms that HOXA13 can use these minimal sites to direct gene expression, confirming their capacity to function as gene-regulatory elements. Values represent average detected luciferase activity after normalization for transfection with a *Renilla* luciferase reporter±standard error.

Because *Tie2 and Foxf1* are strongly expressed in the placental labyrinth and mice lacking TIE2 or FOXF1 exhibit vascular defects most similar to those present in *Hoxa13* mutant labyrinths, we hypothesized that HOXA13 directly regulates *Tie2* or *Foxf1* to facilitate labyrinth vascular development [8], [39], [64]–[70]. Testing this hypothesis, we first examined whether HOXA13 binds the promoters for *Tie2* or *Foxf1 in vivo* using a HOXA13 antibody to immunoprecipitate HOXA13-DNA complexes present in labyrinth chromatin (Figure 10A). Previous characterization of the HOXA13 antibody confirmed that it can bind both wild type and mutant HOXA13 proteins and facilitate chromatin immunoprecipitation (ChIP) of HOXA13-bound gene regulatory elements [71]–[73]. PCR analysis of the first 3000 base-pairs (bp) (−3000 to +1) of the *Tie2* and *Foxf1* promoters revealed a single region in each locus bound by HOXA13 in wild type (not shown) and heterozygous mutant placental labyrinths (Figure 10A and 10B). In contrast, the homozygous mutant HOXA13 protein, which lacks its DNA binding domain, failed to associate with the same promoter regions (Figure 10A). Parallel ChIP assays did not detect HOXA13 association with the promoters of other pro-vascular genes mis-expressed in the mutant labyrinth including *CD36, Caveolin-1*, and *Neuropilin-1* (data not shown).

Sequence analysis of the immunoprecipitated *Tie2* and *Foxf1* promoter regions confirmed the presence of several of the recently identified HOXA13 binding sites (Figure 10B) [71]. Next, using an electrophoretic mobility shift assay (EMSA), the HOXA13 DNA binding domain was confirmed to bind the promoter regions detected by the ChIP assay in a concentration-dependent manner (Figure 10C). Quantitation of HOXA13's affinity for the *Tie2* and *Foxf1* ChIP-positive regions using fluorescence polarization anisotropy revealed high affinity for the binding sites present in *Tie2* (K_d_ = 27±1.4 nM and 22 nM±1.6 nM) and *Foxf1* (K_d_ = 48±4 nM) compared to a control sequence lacking the HOXA13 binding site (K_d_ = 250±22 nM) (Figure 10D and 10E).

Next, the capacity of HOXA13 to regulate gene expression through the 140 base-pair *Tie2* and 121 base-pair *Foxf1* ChIP-positive DNA fragments was examined (Figure 10F). The pGL3-Basic vector was selected for this analysis based on previous studies that confirm its capacity to assess promoter/enhancer activity *in vitro*, including previous characterizations of HOXA13's capacity to regulate transcription from minimal promoter elements [72], [74]–[78]. In the absence of the *Tie2* or *Foxf1* DNA elements, the empty pGL3-basic luciferase plasmid exhibited only a minor increase in luciferase expression when co-transfected with a *Hoxa13* expression plasmid (Figure 10F). Similarly, the same luciferase vector containing either the *Tie2* or the *Foxf1* ChIP-positive regions also exhibited minimal luciferase expression in the absence of HOXA13 (Figure 10F). Co-transfection with a *Hoxa13* expression vector stimulated luciferase expression from these minimal promoter elements resulting in low but significant increases in normalized luciferase expression: 3.7 fold for *Tie2* and 3.2 fold for *Foxf1* (Figure 10F).

## Discussion

### HOXA13 Function Is Co-Opted in Labyrinth Vascular Endothelia

While the timing of *Hoxa13* expression at E7.75 was surprising for a 5′ *Hox* gene, its localization to the allantoic bud is consistent with the later functions of HOXA13 in the gut, where the contributing posterior allantoic bud mesoderm receives patterning instructions from the 5′ HOX proteins [5], [28], [29], [79]–[83]. The vascular-specific expression of *Hoxa13* appears to be restricted to the placental labyrinth, genital tubercle, and umbilical arteries; structures whose ontogeny can be linked to tissues within the posterior embryo [28],[29],[73],[84]. Here, the mesenchymal functions of HOXA13 during genitourinary development appear co-opted to facilitate vascular specification of the labyrinth endothelia [28],[29],[73],[80],[84]. HOXC13 also exhibits co-opted function in the hair follicle, suggesting that the development of specialized structures may utilize the co-opted functions of the group 13 HOX proteins [85]. While other Hox proteins such as HOXD3, HOXB3, HOXA5, and HOXD10 have been shown to regulate angiogenesis, our analysis of HOXA13's function in the vascular endothelia represents, to our knowledge, the only group 13 HOX protein functioning in this capacity [86]–[89].

In the absence of HOXA13 function, labyrinth endothelial cell integrity and vessel branching are compromised, resulting in a placental labyrinth incapable of sustaining fetal growth beyond mid-gestation [27]–[29]. Placental insufficiency also causes secondary cardiac defects [49]–[53]. The absence of *Hoxa13* expression in the early cardiac field as well as its absence in the affected heart tissues supports the conclusion that thinning of the ventricular walls in *Hoxa13* mutants is a secondary defect caused by placental insufficiency.

### Are HOXA13-Deficient Endothelia Assuming a Different Cell Fate?

The over-expression of non-capillary endothelial genes in *Hoxa13* homozygous mutant labyrinths raises an intriguing possibility that the vascular endothelia are trans-differentiating towards a sinusoidal or lymphatic phenotype. Indeed, the genes exhibiting the highest degree of over-expression in *Hoxa13* mutant placentas are either elevated during lymphatic re-specification or have discrete functions in sinusoidal and/or lymphatic endothelia including: *Lyve-1, Igfbp3, Selenbp1,* and *Ednrb* (Table 2) [54], [56]–[58], [90]–[92]. Moreover, the rounded phenotype of *Hoxa13* mutant EC is also more similar to sinusoidal EC, although our analysis of the mutant endothelia by TEM did not reveal substantial fenestrations usually present in sinusoidal endothelia [54]. Perturbations in the expression of the transcription factor, PROX1, also promote *Lyve-1* over-expression in vascular endothelia, suggesting some degree of plasticity in endothelial specification [91],[92]. Transformations of sinusoidal endothelia towards a capillary phenotype have also been induced by selenium, thus the over-expression of the selenium binding protein in the mutant endothelia (*Selenbp1*), could alter endothelial specification by limiting selenium bioavailability[54].

Alternatively, it is possible that the over-expression of LYVE1 in *Hoxa13* homozygous mutant EC could affect the uptake and degradation of matrix hyaluronan, impacting endothelial cell adhesion [90], [93]–[97]. While mice lacking LYVE1 do not exhibit defects in the placental labyrinth, its over-expression in *Hoxa13* mutant EC may influence how these cells interact with the underlying basement membrane resulting in the rounded appearance and perturbations in EC migration [92]. Arguing against this possibility is the result that *Hoxa13* homozygous mutant EC are competent to migrate during angiogenesis, suggesting that LYVE1 over-expression in the mutant EC is a consequence of the loss of endothelial specification. Finally, the over-expression of the metalloprotease, *Bmp1*, could also contribute to the loss in labyrinth vascularization as BMP1 cleaves placental PROLACTIN to produce a potent angiogenic inhibitor [55].

### Loss of HOXA13 Function Does Not Affect Trophoblast Development

Previous studies of labyrinth development suggest that signals from the trophoblast lineages participate in labyrinth formation and vascularization [5], [13], [22], [50], [51], [53], [62], [63], [98]–[102]. The normal expression of trophoblast genes such as *Gcm1, Id1, Id2* (chorionic); *Tpbp* (spongiotrophoblast); and *Esx1* (labyrinth trophoblast) in *Hoxa13* mutant placentas suggests that trophoblast tissues are not affected by the loss of HOXA13 function. Instead, HOXA13 appears to be functioning in the vascular endothelia where it regulates pro-vascular genes necessary for labyrinth vascular development. Similar labyrinth defects are also seen in mice lacking WNT2 and HGF which appear to be predominantly expressed in the allantoic mesoderm and its derivatives [103],[104]. Finally, the normal expression of trophoblast-specific genes also suggests that tetraploid chimeras consisting of a wild type tetraploid embryo and *Hoxa13* homozygous mutant embryonic stem cells would not be effective in rescuing the labyrinth defects [1],[105].

### HOXA13 Coordinates Pro-Vascular Gene Expression To Facilitate Labyrinth Formation

In *Drosophila*, HOX proteins such as UBX regulate the formation of specific structures by controlling genes at multiple levels of a developmental cascade [106]–[108]. Our analysis of HOXA13 function during limb, genitourinary, and placental development suggests a similar mode of gene regulation where the combinatorial functions of direct and indirect target genes are coordinated to facilitate the formation of specific tissues and structures [28], [71]–[73],[80]. Evidence for this coordination is seen in the labyrinth endothelia where genes necessary for cell adhesion and vascular branching are concomitantly affected by the loss of HOXA13 function including: *Neuropilin-1, Enpp2, Lyve1, Caveolin-1, Foxf1*, and *Tie2*, resulting in hypomorphic levels of the provascular factors necessary for labyrinth vascular development.

The binding of HOXA13 to the *Tie2* and *Foxf1* gene regulatory elements in vivo and the reduction of *Tie2* and *Foxf1* expression in *Hoxa13* mutant labyrinths suggest that these pro-vascular genes are direct targets of HOXA13 in the labyrinth endothelia. Indeed, the loss of function phenotypes associated with *Tie2* and *Foxf1* are consistent with the endothelial defects in *Hoxa13* mutant labyrinths. Mice lacking TIE2 exhibit lethality by E10.5 due to a severe lack of angiogenic branching and remodeling of the primary vascular network [39],[70],[109].

*Foxf1* is also essential for placental development [21],[110]. Mice lacking *Foxf1* exhibit mid-gestational lethality resulting from defects in the patterning and vascularization of extra-embryonic tissues [20]. Interestingly, *Foxf1* haploinsufficiency also affects vascular integrity, causing hemorrhaging in the lung and foregut, suggesting that reduced *Foxf1* in the *Hoxa13* mutant labyrinth may be sufficient to affect EC integrity and vessel branching [66]. Similarly, mice lacking ENPP2 also exhibit defects in allantois, yolk sac, and embryonic vessel formation, suggesting that this previously identified HOXA13 target gene may function to mediate labyrinth vascularization.

### Role of HOXA13 Indirect Target Genes

Because the *Neuropilin-1, Caveolin-1,* and *CD36* promoter regions were not detected by the HOXA13 ChIP assay, we are presently classifying these genes as indirect targets of HOXA13. Developmentally, perturbations in *Neuropilin-1, Caveolin-1*, and *CD36* expression are also consistent with the *Hoxa13* mutant labyrinth defects. NEUROPILIN-1, a receptor for VEGF, PlGF-2, and VEGF-B, is essential for endothelial migration and proliferation, and mice lacking this protein die at mid-gestation from vascular and heart defects [14], [15], [111]–[113]. The caveolae-associated molecules *CD36* and CAVEOLIN-1 also modulate cell mobility and permeability, angiogenesis, and intracellular trafficking, and *caveolin-1* knockout mice exhibit decreased vascular tone and decreased angiogenic responses to exogenous stimuli [114]–[117].

### Labyrinth Formation Requires Non-Sprouting and Sprouting Angiogenesis

Factors regulating sprouting and non-sprouting angiogenesis are required for labyrinth development. Initially, non-sprouting angiogenesis regulated by factors such as CYR61 are required to produce the network of chorionic plate vessels (Figure 2) [1],[118]. While *Hoxa13* and *CYR61* homozygous mutants both exhibit reductions in labyrinth vascularization, the vascularization defects are more severe in CYR61 mutants, a consequence of the earlier developmental function of CYR61 which regulates the formation of chorionic plate vessels whose subsequent branches form the primary vessels of the vascular labyrinth (Figure 2) [118]. Similarly, defects in labyrinth vascularization have also been attributed to perturbations in NOTCH/DELTA signaling [41], [119]–[121]. In particular the loss of *Notch1* function or its target genes, *Hey1* and *Hey2*, cause a complete loss of angiogenic sprouting necessary to form the vascular labyrinth [41], [119]–[121]. Similar to HOXA13, the NOTCH ligand, *DLL4*, appears to be expressed in the umbilical arteries, as well as the developing chorionic plate vessels and invading labyrinth branches [119]. In these tissues, haploinsufficiency of DLL4 was sufficient to cause gestational lethality, a consequence of the regression of the chorionic plate vessels which integrate the developing umbilical vessels to the placental labyrinth [119]. While this phenotype is substantially different from the labyrinth defects in *Hoxa13* homozygous mutants the similarities in the expression domains of *Hoxa13, Notch1*, and *Dll4* raise the possibility that HOXA13 and activated Notch receptors function through common factors to promote the angiogenic processes necessary for labyrinth development. Factors such as NEUROPILIN-1 may be a common link between HOXA13 and NOTCH/DELTA signaling as this essential pro-angiogenic molecule is down-regulated in *Hoxa13* (this work), *Notch1*, and *Hey1/Hey2* mutants [41].

The formation of a functional placenta is one of the most critical steps in human and mouse intrauterine development. In this study we have identified a role for HOXA13 in the formation of this vital organ. These findings suggest that HOXA13 regulates a series of genes in the vascular endothelia that are necessary for adhesion and vessel branching, providing a functional explanation for the mid-gestational lethality exhibited by *Hoxa13* mutant mice. More importantly, these findings provide a novel genetic pathway to consider when characterizing pathologies of the placenta or placental evolutionary ontogeny.

## Materials and Methods

### *Hoxa13-GFP* Mutant Mice

All animal care and handling was done following an approved institutional animal protocol. Mice used in this study were from the *Hoxa13-GFP* line, previously described [28]. *Hoxa13* mutant embryos were derived from heterozygous intercrosses as described [28],[80]. Timed matings were used to establish embryonic gestational age and are depicted in embryonic days (E) where E0.5 represents the first day of vaginal plug detection. The *Hoxa13* mutant allele encodes a fusion protein where the last 34 amino acids of HOXA13, which encodes the DNA binding domain, have been replaced with an EGFP reporter as described [28],[73]. The nuclear localization, protein turnover, and tissue-specific expression of the HOXA13-GFP protein were similar to the wild type protein [28]. The HOXA13-GFP fusion protein produces a robust fluorescent signal. Analyses examining endogenous HOXA13-GFP localization are labeled as HOXA13-GFP. Because E11.5–13.5 placentas exhibit high autofluorescence in the GFP emission spectra it was necessary to use a GFP antibody (AB3080, Chemicon) to visualize the co-localization of the HOXA13-GFP with candidate target gene proteins. Characterizations of HOXA13-GFP expression using the GFP antibody are denoted as α-GFP in the figures. A Texas-red labeled secondary antibody (Jackson Immunological) was used to detect the localization of the GFP antibody.

### Immunohistochemistry and In Situ Hybridization

Placentas were dissected in cold 1× PBS and fixed 3 hours to overnight in 4% paraformaldehyde/PBS at 4°C rocking. For frozen sections, placentas were treated with a 10–30% sucrose/PBS gradient, embedded in OCT (Tissue-Tek, Inc), and stored at −80°C. Frozen OCT-embedded placentas were sectioned at 17–20 µm using a Leitz Kryostat 1740 and mounted on Superfrost plus slides (Fisher). Immunohistochemistry (IHC) and *in situ* hybridization (ISH) experiments were carried out as previously described [73],[80]. The *Tpbp* riboprobe plasmid was kindly provided by Dr. James Cross (University of Calgary).

For whole placenta IHC, placentas were bisected with a double-edged razor blade and fixed in 4% paraformaldehyde overnight at 4°C. After fixation the bisected placentas were dehydrated with a 4∶1∶1 methanol-DMSO-peroxide solution and incubated in primary and secondary antibodies overnight followed by extensive PBST washes. The following antibodies and dilutions were used: PECAM-1 for section (#550274, BD Pharmingen, 1∶200), PECAM-1 for whole placenta IHC (MEC13.3, #553369 BD Pharmingen, 1∶200), GFP (AB3080, Chemicon, 1∶100), Lyve1 (ab14917, Abcam, 1∶200), Tie2 (#MAB1148, Chemicon, 1∶200), ENPP2 (Cosmo Bio, 1∶100).

For co-localization studies, CY5- or Texas Red-labeled secondary antibodies were used as described [73],[80]. To visualize the co-localization of PECAM-1 and LYVE1 in the vascular endothelia, the red CY5 signal detecting the distribution of the PECAM-1 antibody was pseudo-colored green using the Laser Sharp 2000 software (BioRad).

### Transmission Electron Microscopy (TEM)

E11.5 placentas were bisected and immediately immersed in 1.5% glutaraldehyde/1.5% paraformaldehyde with 0.05% tannic acid and 5.0% sucrose in DMEM media for 2 hours on ice, rinsed in several changes of DMEM, then post-fixed in 1.0% OsO_4_ in DMEM for an additional 90 minutes on ice. Fixed tissues were rinsed in several changes of DMEM over 15 minutes, then dehydrated in a graded ethanol series to 100%, rinsed in propylene oxide, and finally infiltrated and embedded in Spurr's epoxy. Ultrathin sections were cut at 80 nm, contrasted with uranyl acetate and lead citrate, and then examined using a Philips 410 TEM operated at 80 KV. E13.5 placentas were fixed by perfusion using 1.5% glutaraldehyde/1.5% paraformaldehyde with 0.05% tannic acid in DMEM, then bisected and immersed in the same fixative for an additional 60 minutes. Post-fixation, dehydration and embedding was identical to the E11.5 embryos described above.

### Morphological Scoring of the Labyrinth Endothelia

E13.5 placentas were sectioned at 1 µm, mounted on slides, and counterstained with toluidine blue with basic fushin and the sections containing the labyrinth regions were photographed. For each placental sample, seven to ten photographs representing nearly the complete vascular labyrinth region were taken. Within each photo, fetal labyrinth lumens were identified, and all ECs with a visible nucleus were counted and their morphology scored as either normal (smooth elongated EC layer and lumen), intermediate (some cell rounding or irregular shape), or abnormal (cell detachment, edema, diminished cell body). Three wild type placentas (25 photos; 1510 endothelial cells), five *Hoxa13* heterozygous placentas (38 photos; 1938 ECs), and five *Hoxa13* homozygous mutant placentas (39 photos; 1301 ECs) were analyzed. The average percentage of each cell morphology was calculated and plotted with their standard deviations using Excel (Microsoft).

### Quantitative Analysis of Placenta Labyrinth Thickness and Vascular Branching

Placental labyrinth thickness was measured in 8 independent E12.5 Hoxa13 heterozygous and homozygous mutant placentas. The placentas were fixed, OCT embedded, and 20 µm sections were hybridized with the *Tpbp* or *Hoxa13* (not shown) riboprobes to identify the labyrinth and overlying spongiotrophoblast regions. Digital photographs were taken from two separate placental sections that represent middle regions of each placenta, using a Leica DML light microscope with a 10× objective and a Q-Imaging camera. Using the NIH Image J software, labyrinth thicknesses (in micrometers) were measured from the labyrinth-spongiotrophoblast border to the chorionic plate at five independent points along the placental section. For each point, three to six independent measurements were taken. The average thickness and standard deviation was calculated using Excel (Microsoft) and plotted using Sigmaplot 9.0 (Systat).

Labyrinth vessel branching was quantitated using a modified grid analysis of the labeled labyrinth vessels as described [122]. Hemisected E13.5 placentas were fixed in 4 percent paraformaldehyde and processed for whole mount immunohistochemistry using the PECAM-1 antibody as described earlier. The hemisected placentas were photographed using a Leica MZ-12.5 stereoscope fitted with a Nikon Coolpix 990 digital camera at 4× magnification. Placenta photographs were resized to 4×2.5 inches using Adobe Photoshop CS3. Three hemisected placentas were characterized for each genotype. A 400 mm^2^ (20 mm×20 mm) grid was placed at six independent locations in the labyrinth region of the placental photographs and the PECAM-1 labeled vessels branches present in the grid were counted. The average number of vessel branches and their standard deviations were calculated using Excel (Microsoft) and plotted using Sigmaplot 9.0 (Systat).

### Angiogenesis Assay

Primary umbilical arteries entering the placenta (E13.5 embryos) were dissected free using sterilized tungsten needles. The dissected vessels were cut into 1–3 mm sections using sterilized microvasculature scissors (Fine Science Tools) and embedded into 1 percent type I collagen (BD Biosciences) containing 2.3 mg/ml sodium bicarbonate, and 2× enrichment of EGM-2 MV base media (Lonza, Walkersville, MD) as described [123]. Four-well chambered microslides (NUNC) were used for the embedding and culture of the vessel sections. After 10 minutes of incubation at 37 degrees Celsius, the chambered slides were filled with complete 1× EGM-2MV media supplemented with 5 percent fetal bovine serum, and the Single-Quot® growth factor/antibiotic cocktail containing hydrocortisone, hFGF-B, VEGF, IGF, ascorbic acid, EGF, gentamicin, and amphotericin-B as described by the manufacturer (Lonza, Walkersville, MD). After five days, the cultured umbilical arteries were fixed overnight in 4 percent paraformaldehyde/PBS at 4 degrees Celsius. After fixation the cultured vessels were rinsed with 1× PBS containing 1% Triton X 100, and characterized for HOXA13-GFP and PECAM-1 expression as described [73],[80].

### Quantification of the Ventricular Wall Thickness

Three separate E13.5 Hoxa13 heterozygous control and homozygous mutant embryos were fixed, paraffin-embedded, sectioned, and stained with hematoxylin and eosin using standard histological techniques as described [73]. The heart regions from the sectioned embryos were photographed using a Leica DML compound microscope and a Q-Imaging digital camera. The cellular thickness of each ventricular wall was determined by counting the number of hematoxylin-stained nuclei present in 6–10 perpendicular lines drawn from outer edge of the left ventricular wall to the trabeculae as shown in Figure 8. The average cell number and standard deviation was calculated using Excel (Microsoft).

### Microarray and Statistical Analysis

E13.5 placentas were dissected and the isolated umbilical vessels, embryonic labyrinth, and spongiotrophoblasts tissues were used as the source for the embryonic placental RNA. Tissues were dissected in RNAlater (Ambion), flash-frozen in liquid nitrogen, and stored at −80°C. The RNA STAT-60 (CS-110, Tel-Test, Inc.) and RNeasy Micro Kit (QIAGEN) systems were used for RNA extraction, following the manufacturer's protocol. RNA quality was assessed using an Agilent Bioanalzyer at the OHSU Affymetrix Microarray Core facility (AMC), UV spectroscopy, and agarose gel electrophoresis. Three RNA samples of like genotype were pooled for each microarray analysis. Twelve independent microarray hybridizations (6 *Hoxa13* homozygous mutant, 6 wild type) were performed using the MOE430A and B microarrays (Affymetrix). MAS 5.0 software (Affymetrix) was used by the AMC to collect and normalize the array data. Statistical analysis of the microarray data sets was performed by the Biostatistics and Bioinformatics Shared Resources Core Facility at OHSU. A two-factor analysis of variance (ANOVA) was used to determine the false discovery rate and to compare transcript signal intensities between wild type and homozygous mutant placental tissues.

### Collection of Placenta Endothelial Cells for qRTPCR Analysis

E13.5 placental EC were isolated by dissecting fresh placentas as in the microarray method. Two to three placentas of the same *Hoxa13* genotype were combined. Samples were treated with 0.2% Collagenase Type IV (#17104-019, Gibco) at 37°C for 30 minutes, with occasional shaking. Tissue was then transferred to a Netwells Dish (Costar) and treated with digestion media (0.1% Trypsin/EDTA, 0.2% Collagenase IV, in PBS) for 30 minutes at 37°C using gentle pipetting to dissociate the tissue. Magnetic beads (Dynabeads M450, DYNAL Inc) were coated with PECAM-1 antibody (MEC 13.3, #553369 BD Pharmingen) as described by the manufacturer. 2.5×10^6^ cells were dispensed into 0.7 ml micro-tubes with the Dynabeads-PECAM1 antibody (3× more beads than cells), and incubated for 1 hour at 4°C on rotating platform. The bead-antibody-cell complexes were isolated using a magnet and the remaining cell supernatant was collected as a PECAM(-) control. The cell complexes were gently washed with 0.1% BSA/PBS and collected for RNA extraction.

RNA was extracted from both the PECAM+ and PECAM− cell samples using the RNA Stat-60 (CS-110-Tel-Test, Inc.) and RNeasy Micro Kit systems (QIAGEN) and treated with DNase I (Promega) to remove genomic DNA. RNA quality was analyzed by UV spectroscopy and agarose gel electrophoresis. One microgram of RNA was used for cDNA synthesis using the Superscript First-Strand Synthesis system (Invitrogen). The SYBR-green based system of gene expression detection was utilized to quantify the relative levels of control and placenta-specific genes in each cDNA sample. To distinguish amplicons generated from genomic DNA, PCR primers were designed to span an intron-exon boundary. Melting curves were empirically determined for each primer set to establish precise temperature for data collection. Reaction amplifications were performed using a 1× SYBR Green PCR Master Mix as described by the manufacturer (Applied Biosciences). QRT-PCR reactions were performed in triplicate, and each gene primer set was tested against a minimum of four cDNA samples derived from independent RNA isolates. All qRT-PCR reactions were performed using an Applied Biosystems Model 7700 Sequence Detector. The primer sequences for the control genes (*gapdh*, *actin*, and *Pecam-1*) and candidate genes are as follows: *MageL-2*, For-5′-GAACCCACGACCAGAACC-3′, Rev-5′-CTTAGTGTTGGCACGGTTGA-3′ (132 bp, Ex1); *Necdin*, For-5′-AACGCTTTGGTGCAGTTTCT-3′, Rev-5′- AACACTCTGGCGAGGATGAC-3′ (134 bp, Ex1); *Adrb1*, For-5′-TGGTACGTGTTGGTGAAGGA-3′, Rev-5′- AAGTCCAGAGCTCGCAGAAG-3′ (100 bp, Ex1); *Enpp2*, For-5′-CCGACCTGACAATGATGAGA-3′, Rev-5′- AAATCCAAACCGGTGAGATG-3′ (120 bp, Ex24–25); *Tie2*, For-5′-TGAGGACGCTTCCACATTC-3′, Rev-5′- CAACAGCACGGTATGCAAGT-3′ (104 bp, Ex13–14); *Foxf1*, For-5′-GCAGCCATACCTTCACCAA-3′, Rev-5′-GCCATGGCATTGAAAGAGA-3′ (126 bp, Ex1–2); *Neuropilin-1*, For-5′-TGTCCTGGCCACAGAGAAG-3′, Rev-5′- CCAGTGGCAGAATGTCTTGT-3′ (115 bp, Ex12–13); *Caveolin-1*, For-5′-GGGAACAGGGCAACATCTAC-3′, Rev-5′-ACCACGTCGTCGTTGAGAT-3′ (136 bp, Ex1); *CD36*, For-5′-GAGTTGGCGAGAAAACCAGT-3′, Rev-5′-GTCTCCGACTGGCATGAGA-3′ (143 bp, Ex3); *Lyve-1*, For-5′-AGCCAACGAGGCCTGTAA-3′, Rev-5′-CACCTGGGGTTTGAGAAAAT-3′ (150 bp, Ex 2–3); *Pecam-1*, For-5′-CCAGTGCAGAGCGGATAAT-3′, Rev-5′-GCACCGAAGTACCATTTCAC-3′ (148 bp, Ex7–8); *Actin*, For-5′-CCTGCCATGTATGTGGCTAT-3′, Rev-5′-CTCATAGATGGGCACGTTGT-3′ (114 bp, Ex3–4); *Gapdh* For-5′-CACTGCCACCCAGAAGACTGT-3′, Rev-5′-GGAAGGCCATGCCAGTGA-3′ (147 bp).

### Chromatin Immunoprecipitation (ChIP)

Whole placentas were cut into 2 mm sections and fixed for 60 seconds in a 1% formaldehyde solution followed by treatment with 1M glycine/PBS/protease inhibitors. The fixed tissues were incubated 10 minutes in SDS lysis buffer (Upstate Biotech) and homogenized using disposable plastic dowels. The released chromatin was sheared at 4°C using a Biorupter sonicator (CosmoBio, Japan). Proper sonication was confirmed by agarose gel electrophoresis. A ChIP assay kit (Upstate Biotech) was used to isolate HOXA13-DNA fragments from sheared placental chromatin. Prior to ChIP, the presence of the HOXA13 protein in the sheared chromatin was confirmed by western-immunoblot using the HOXA13 antibody previously described [72]. Chromatin produced by the HOXA13-ChIP assay was examined for the *Foxf1* and *Tie2* promoter sequences containing the previously described HOXA13 binding sites [71] using PCR and 30 cycles of 94 degrees Celsius for 30 seconds, 54 degrees Celsius for 30 seconds, and 72 degrees Celsius for 30 seconds. Primers flanking the potential HOXA13 binding sites were selected using the genomic sequences listed for *Foxf1* (Ensembl:MUSG00000042812) and *Tie2* (Ensembl:MUSG00000006386). The PCR primers were: *Foxf1*#1 (−2368 to −2231, 138 bp), For-5′-TGAGGTACAGCCCAGAGTCC-3′, Rev-5′-CACACCCCCAAGTTTTCTTC-3′; *Foxf1*#2 (−1284 to −1163, 122 bp), For-5′-CGCGGGCTTCTCTACTCTTA-3′, Rev-5′-CCTTTTACAAGCGCAGGTTC-3′; *Tie2*#1 (−2413 to −2273, 141 bp), For-5′-GGGAAGGGGAGTGGATAACA-3′, Rev-5′-CTAATCCCAGCCCTGCTGTA-3′; *Tie2*#2 (−859 to −702, 158 bp), For-5′-CTTCCTGTGCCAAGTTCTCC-3′, Rev-5′- GACCAGATTCCACAGCCATT-3′. Each ChIP assay was performed at least two times using unique placenta samples in order to confirm the ChIP results.

### Electrophoretic Mobility Shift Assays and Luciferase Assays

The HOXA13 DNA-binding domain peptide was purified and gel shifts performed as previously described [72]. The *Foxf1* and *Tie2* primers described in the ChIP assay were used to amplify the genomic regions for the EMSA assay. The amplified PCR products were isolated using the QIAquick Gel Extraction Kit (Qiagen), quantified by UV spectroscopy, and sequenced to confirm the correct gene promoter sequence. PCR amplicons were radiolabeled using γ-^32^P dATP (3000 Ci/mmol; 1Ci-37 GBq) and T4 polynucleotide kinase as described by the manufacturer (Promega). EMSA assays were performed using the Gel Shift Binding System following manufacturer's protocol (Promega).

The 121 bp *Foxf1* and 140 bp *Tie2* DNA elements identified by ChIP to be bound by HOXA13 in the labyrinth vascular endothelia were evaluated *in vitro* for their capacity to facilitate gene expression in the presence of HOXA13. Based on the small size of these ChIP-positive DNA sequences as well as our previous characterization of HOXA13's relatively low *in vitro* transcriptional activity, a promoter- and enhancer-less luciferase reporter plasmid, pGL3-Basic (Promega), was selected for this analysis [65],[66]. The pGL3-Basic vector has been previously shown to measure minimal promoter activity with high reproducibility [71], [72], [74]–[77],[124]. Luciferase assays and cell culture were performed as previously described [71],[72]. The NG108-15 cell line (ATCC#HB-12317) was selected for the luciferase assay based on the absence of endogenous HOXA13 expression [124]. Transfections were performed in 12 well plates (Costar) using 2 µg of the *Tie2* or *Foxf1* luciferase vectors, 0.25 µg pRL-CMV *Renilla*, and 0.5 µg pCAGGS-*Hoxa13* or empty pCAGGS control plasmid per well as described [71]. Cell lysates were processed to detect luciferase activity using the Dual-Glo Luciferase Assay System (Promega) in OptiPlate-96F black plates (Packard) as described (Promega) [72]. Luciferase activity was detected using a Packard Fusion Microplate Analyzer (Perkin Elmer), wells were read 3 times for 1 sec each and averaged. Three replicates of each transfection were performed and each transfection assay was repeated 3 times. Results were normalized for Renilla expression and averaged. The averaged data and standard errors were plotted using SigmaPlot 9.0 (Systat).

### Fluorescence Anisotropy

Quantification of HOXA13 affinity for the binding sites present in *Foxf1* and *Tie2* was determined by fluorescence polarization (FP) anisotropy as described [71]. Fluorescein-labeled self-annealing hairpin oligonucleotides specific for the binding sites in the ChIP-positive regions of *Tie2* and *Foxf1* were synthesized by Integrated DNA Technologies (Coralville, IA): *Tie2*#1: 5′-ctgtaattaaataccccccgtatttaattacag-3′; *Tie2*#2: 5′-catttaataaaaaccccccgtttttattaaatg-3′; *Foxf1*: 5′-cttattattaaaggccccccctttaataataag-3′; TGAC control: 5′-tgactgactgactgccccccagtcagtcagtca-3′. 1 nM of the annealed fluorescein-labeled oligonucleotides was incubated with 0–200 nM HOXA13-DBD peptide at 15 degrees Celsius and the FP values were measured using the Beacon 2000 Fluorescence Polarization System (Invitrogen). The detected millipolarization values (mP) were plotted using a non-linear least squares fit iteration as described [71]. Each data point represents an average (±standard error) of 3 or more independently derived mP values for each concentration of HOXA13-DBD peptide.

## Supporting Information

Figure S1. Developmental analysis of labyrinth formation and pro-vascular gene expression. (A, B) PECAM-1 immunostaining revealed no difference in labyrinth vascular bed initiation between *Hoxa13* control (+/−) and homozygous mutants at E10.5. (C–F) *Hoxa13* homozygous mutants exhibit a decrease in primary vascular branching as early as E11.5 compared to wild-type controls. (E) and (F) represent higher magnification images of the PECAM-1 stained vessels depicted in (C) and (D), respectively. (G–J) TIE2 expression is reduced in the labyrinth region of *Hoxa13* homozygous mutants compared to heterozygous controls at both the initial stages of labyrinth formation at E10.5 and during primary vessel branching at E12.5. (K–N) *Hoxa13* homozygous mutant placental labyrinths exhibit a consistent increase in LYVE1 immunostaining compared to controls at E11.5 and E12.5.
(1.50 MB TIF)
