# Supplementary figures and images for: HOXA13 Is Essential for Placental Vascular Patterning and Labyrinth Endothelial Specification
Source: PLoS Genet. 2008 May 16;4(5):e1000073. doi: 10.1371/journal.pgen.1000073 (PMC2367452; doi:10.1371/journal.pgen.1000073)

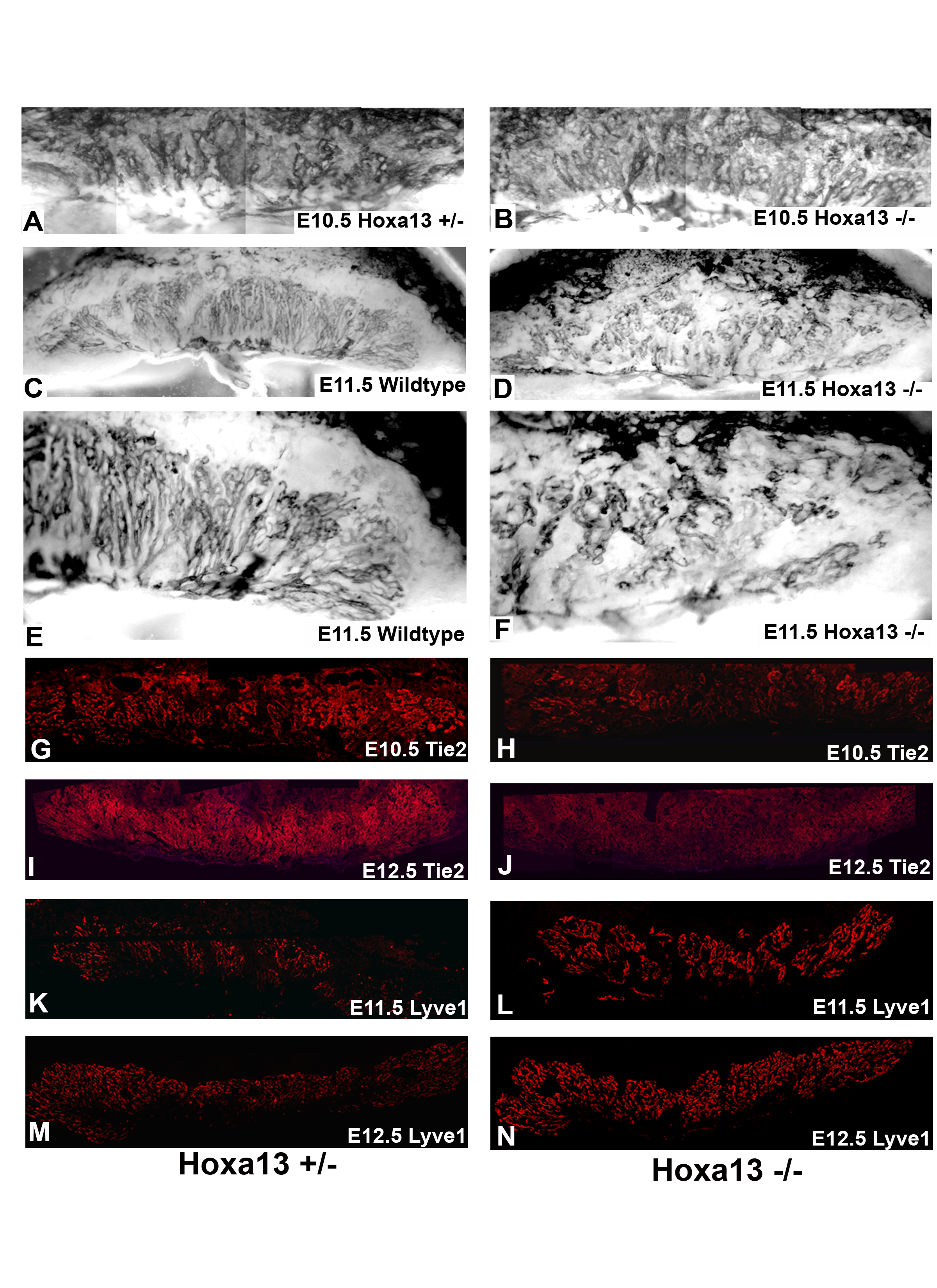

Supplement: Figure S1. — Developmental analysis of labyrinth formation and pro-vascular gene expression. (A, B) PECAM-1 immunostaining revealed no difference in labyrinth vascular bed initiation between Hoxa13 control (+/−) and homozygous mutants at E10.5. (C–F) Hoxa13 homozygous mutants exhibit a decrease in primary vascular branching as early as E11.5 compared to wild-type controls. (E) and (F) represent higher magnification images of the PECAM-1 stained vessels depicted in (C) and (D), respectively. (G–J) TIE2 expression is reduced in the labyrinth region of Hoxa13 homozygous mutants compared to heterozygous controls at both the initial stages of labyrinth formation at E10.5 and during primary vessel branching at E12.5. (K–N) Hoxa13 homozygous mutant placental labyrinths exhibit a consistent increase in LYVE1 immunostaining compared to controls at E11.5 and E12.5. (1.50 MB TIF) [file pgen.1000073.s001.tif]
